# The relationship between ankle dorsiflexion range of motion and reactive postural control mechanisms in response to anterior perturbations

**DOI:** 10.1371/journal.pone.0362395

**Published:** 2026-11-12

**Authors:** Mosa Pashaei, Hadi Ramezanloo, Mohamadreza Hatefi

**Affiliations:** 1 Department of Sport Injuries and Corrective Exercises, Faculty of Physical Education and Sport Science, Allameh Tabataba’i University, Tehran, Iran; 2 Department of Motor Development, Faculty of Physical Education and Sport Sciences, Islamic Azad University, Tehran, Iran; King Khalid University, SAUDI ARABIA

## Abstract

Adequate ankle dorsiflexion range of motion (DF-ROM) is essential for generating sufficient corrective torque during an ankle strategy; however, limitations in dorsiflexion may mechanically constrain this response, potentially leading to earlier reliance on hip or stepping strategies. Although indirect evidence links dorsiflexion capacity to dynamic balance performance, it remains unclear whether restricted dorsiflexion influences the selection of reactive postural control strategies in healthy young adults. Twenty active male participants were recruited for this study. Ankle DF-ROM was measured via the weight-bearing lunge test and categorized as either normal (≥20°) or restricted (<20°). Reactive postural control was assessed in response to a single unexpected anterior perturbation administered by a custom-engineered movable platform, with the perturbation load normalized to participants’ body mass. Sagittal-plane kinematics were recorded at 60 Hz and independently evaluated by two blinded raters to classify the initial balance recovery response into ankle, hip, or stepping strategies based on established criteria. Statistical analysis revealed no significant association between ankle DF-ROM category and the type of reactive postural control strategy employed following anterior perturbations. Pearson’s chi-square test indicated no significant relationship between dorsiflexion status and balance strategy selection (*χ*^2^(2) = 0.826, p = 0.662). Due to low expected cell counts, Fisher’s exact test was also conducted, corroborating the absence of a significant association (two-sided p = 0.835). The distribution of postural strategies did not differ substantially between participants with normal and restricted dorsiflexion. In healthy young adults exposed to a standardized anterior perturbation, ankle DF-ROM was not independently associated with reactive postural strategy selection. These findings suggest that the postural demands imposed by the perturbation may have contributed to the predominance of stepping responses and may have reduced the sensitivity to detect an influence of ankle mobility on strategy selection.

## Introduction

The maintenance of upright posture following a sudden displacement of the support surface necessitates rapid sensorimotor integration to realign the body’s center of mass (COM) within the base of support (BOS) [1,2]. In response to minor and gradual perturbations, an “inverted pendulum” mechanism centered around the ankle joint is generally sufficient; corrective torques are produced through a sequential activation pattern progressing from distal to proximal muscles, involving the lower leg, thigh, and trunk [3,4]. Conversely, larger or more rapid perturbations often exceed the stabilizing capacity of the ankle, thereby eliciting a hip strategy characterized predominantly by proximal muscle engagement and trunk movement, or a stepping strategy, which entails repositioning the foot to reestablish the BOS beneath the COM [3,5]. The stepping strategy, involving modifications to the BOS such as forward or backward stepping, is typically employed when fixed-support strategies fail to preserve postural equilibrium [3,5].

Sufficient ankle dorsiflexion mobility is considered essential for effective postural control [6,7]. Limitations in ankle dorsiflexion restrict the forward progression of the tibia over the foot, thereby reducing the moment arm available for generating corrective ankle torque. Individuals exhibiting dorsiflexion deficits frequently compensate by increasing hip flexion and knee movement to maintain balance during weight-bearing activities [8,9]. Such compensatory mechanisms, including stiffer landings characterized by reduced knee flexion, have been associated with elevated ground reaction forces and an increased risk of injury among athletic populations [8,9]. Indeed, restricted ankle dorsiflexion has been recognized as a significant risk factor for lower extremity injuries [9,10]. Conversely, a greater range of dorsiflexion facilitates smoother and deeper squatting and jumping movements; for instance, athletes with enhanced ankle flexibility demonstrate superior squat-jump performance [11]. In older adults, increased dorsiflexion range of motion is predictive of improved dynamic balance and functional mobility [6], while impaired dorsiflexion force control correlates with deficits in gait and balance [12,13].

It can be inferred that healthy adults exhibiting reduced ankle dorsiflexion may demonstrate a diminished capacity to employ an ankle strategy, potentially leading to an earlier transition to hip or stepping strategies when balance is disrupted [14,15]. Consistent with this notion, individuals with chronic ankle instability—a condition frequently characterized by restricted dorsiflexion—often display attenuated ankle-sway responses alongside an increased dependence on hip or stepping responses during balance perturbations [14,16]. In addition to altered postural sway characteristics, individuals with chronic ankle instability have demonstrated impaired reactive balance responses during unexpected perturbations. Recent investigations using externally induced perturbation paradigms have reported delayed neuromuscular activation, altered lower-limb coordination, and reduced capacity to rapidly restore stability following unexpected balance disturbances. These findings suggest that ankle dysfunction may influence not only steady-state balance performance but also the neuromechanical mechanisms underlying reactive postural control [17]. Furthermore, research has identified moderate positive correlations between ankle dorsiflexion range of motion (DF-ROM) and performance on dynamic balance assessments, such as the Y-Balance or Star Excursion tests, among physically active populations [15,18]. These observations imply a functional association between dorsiflexion capacity and postural control proficiency. Although previous studies have demonstrated associations between ankle dorsiflexion ROM and measures of dynamic balance or postural performance, most have relied on functional reach tests, clinical balance assessments, or overall stability outcomes rather than directly examining the biomechanical strategy selected during reactive balance recovery. Consequently, it remains unclear whether limitations in ankle dorsiflexion influence the initial selection of ankle, hip, or stepping strategies following an unexpected perturbation. Addressing this gap may improve our understanding of the mechanical contribution of ankle mobility to reactive postural control.

Therefore, the primary aim of the present study was to determine whether ankle dorsiflexion ROM influences the initial selection of reactive postural control strategies (ankle, hip, or stepping) following an unexpected anterior perturbation delivered using a standardized custom-built perturbation platform. It was hypothesized that individuals exhibiting limited dorsiflexion would demonstrate a reduced reliance on pure ankle strategies and an increased utilization of hip and stepping strategies, relative to those with normal dorsiflexion. Elucidating this relationship may advance our comprehension of the mechanical and neuromuscular factors influencing reactive balance control and contribute to the development of targeted interventions aimed at optimizing performance and preventing injury in physically active populations.

## Methods

### Participants

Recreationally active male individuals (Age: 20.10 ± 5.89 years; Height: 178.86 ± 7.01 cm; Mass: 85.15 ± 13.01 kg; BMI: 24.15 ± 3.41) were recruited through convenience sampling at a local fitness facility. An a priori sample size calculation was performed using G*Power (Version 3.1). The calculation was based on a chi-square test of independence, assuming a large effect size (Cohen’s w = 0.50), a significance level (α) of 0.05, and a statistical power of 0.85. The estimated minimum sample size was approximately 18 participants based on the assumed large effect size (Cohen’s w = 0.50), an alpha level of 0.05, and a statistical power of 0.85. To account for potential participant attrition or incomplete data, 20 participants were recruited. Because the sample size calculation was based on a large effect size, the study may have had limited statistical power to detect small-to-moderate associations and was therefore considered exploratory in nature. To account for potential attrition, an initial cohort of twenty participants was enrolled. Recruitment occurred at Fit gym clubs in Tehran between 05/09/2025 and 28/10/2025. Inclusion criteria stipulated that participants engage in physical exercise of at least light-to-moderate intensity on a minimum of three days per week and have no current lower-extremity pain or functional impairment. Exclusion criteria encompassed any lower-limb injury or surgical intervention within the preceding 6–12 months, neurological or vestibular disorders, or any medical condition that could compromise balance. The study protocol was approved by the Ethics Committee of Allameh Tabataba’i University, Tehran, Iran (Approval No.: IR.ATU.REC.1403.156). All procedures were conducted in accordance with the principles of the Declaration of Helsinki. Written informed consent was obtained from all participants prior to their inclusion in the study.

### Procedures

Participants attended a single testing session at the Gym club, which lasted approximately one hour. Upon arrival, demographic data including age, height, and body mass were collected. Dorsiflexion angle was measured using a digital inclinometer positioned on the anterior aspect of the tibia at the level of the tibial tuberosity during the weight-bearing lunge test (WBLT) [19,20]. Two trials were performed on the dominant limb, and the maximum value was used for analysis. The dominant limb was defined as the participant’s preferred leg for kicking a ball. During this assessment, participants positioned one foot at a predetermined distance from a wall and performed a forward lunge while maintaining heel contact, progressing until maximal dorsiflexion was achieved. The forward lean continued until the heel was on the verge of lifting, indicating the end range of ankle motion. The test was conducted bilaterally; however, classification was based on the maximum value obtained from two trials on the dominant limb. Dorsiflexion angle was quantified either by placing a digital inclinometer on the anterior shin at the tibial tuberosity [20,21]. These measurement techniques are well-established for the WBLT and exhibit high inter- and intra-rater reliability, with intraclass correlation coefficients ranging from approximately 0.80 to 0.99 [19,20]. According to normative data from comparable populations, dorsiflexion was classified as normal if the lunge ankle DF-ROM was equal to or greater than 20°, and restricted if less than 20° [19]. This threshold, although lower than some earlier standards, was selected to ensure a distinct group differentiation. The angle recorded corresponded to the point of maximal comfortable forward lean. All assessments were conducted by a trained examiner to minimize measurement error. Prior to testing, participants completed a standardized 5-minute low-intensity warm-up consisting of light weight-bearing movements and dynamic lower-extremity mobility exercises under the supervision of the examiner. The protocol was specifically designed to prepare participants for testing while minimizing the risk of neuromuscular fatigue. No high-intensity or fatiguing activities were performed before the perturbation assessment.

### Perturbation apparatus and protocol

Reactive postural control was evaluated utilizing a custom-designed anterior perturbation platform. This device comprised two superimposed platforms: a stationary base weighing 50 kg and a movable upper platform weighing 20 kg, each measuring 80 × 50 cm in surface area. The two platforms were separated by eight low-friction bearings, facilitating smooth horizontal translation. The movable platform was connected through a low-height (30 cm) pulley system to a release mechanism loaded with weights. To minimize inter-individual differences in perturbation intensity, a constant 15-kg load was combined with an additional load equivalent to approximately 10% of each participant’s body mass. The same platform configuration, pulley system, release mechanism, and loading procedure were used for all participants, and all perturbations were administered by the same examiner under identical experimental conditions. Upon activation of the release mechanism, the weighted cable produced a sudden anterior translation of the movable platform. Although the platform displacement, velocity, acceleration, and applied pulling force were not directly instrumented or quantitatively measured, the mechanical configuration and loading conditions were standardized across participants. The same platform configuration, pulley arrangement, external load, release procedure, and examiner were used for all participants. Therefore, the perturbation protocol was standardized experimentally; however, its biomechanical magnitude and loading characteristics could not be precisely quantified (Fig 1).

**Fig 1:**
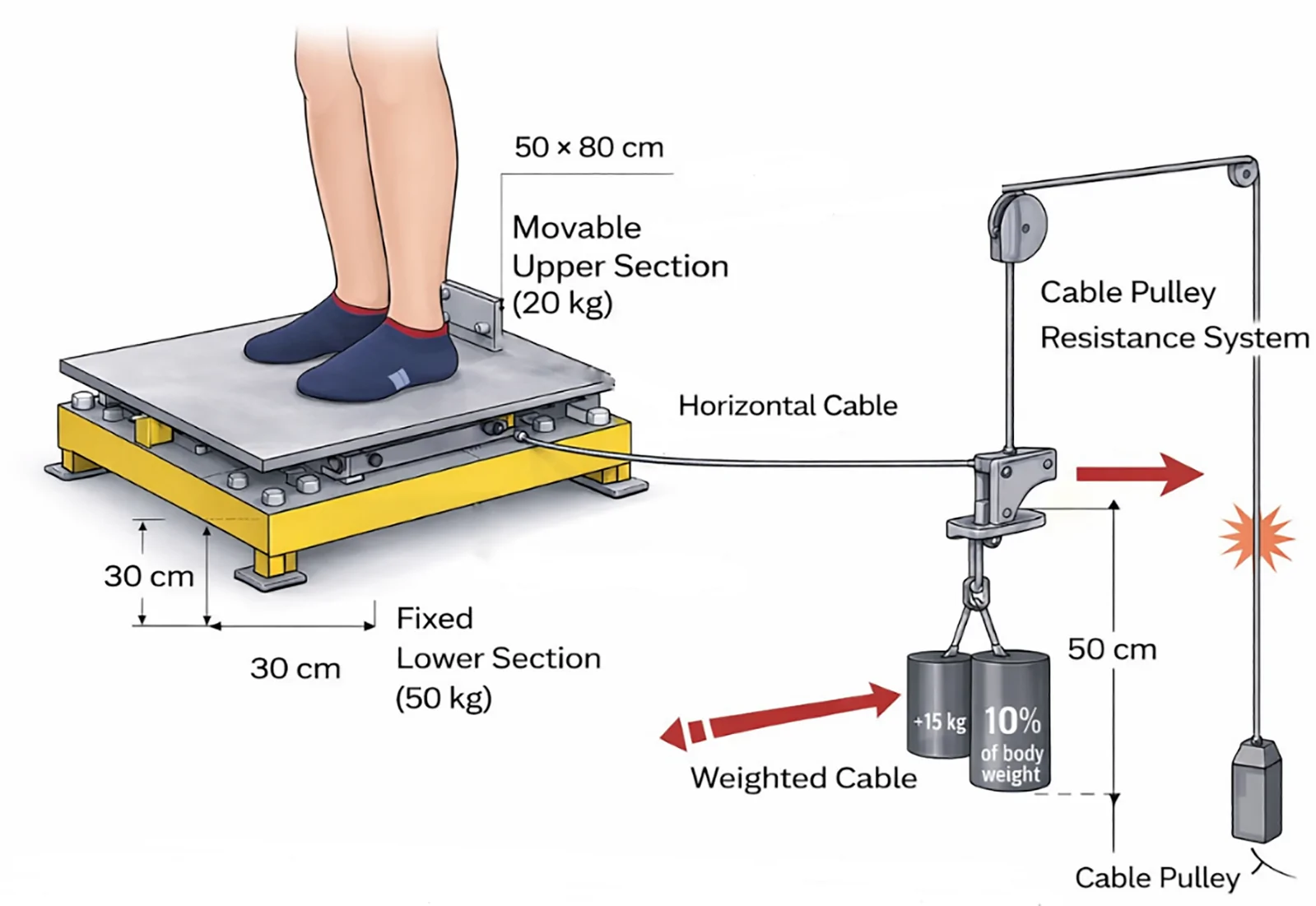
Schematic illustration of the custom-designed anterior perturbation platform used to assess reactive postural control.

To ensure the perturbation was unexpected, participants stood barefoot on the movable platform facing away from the direction of translation, with feet positioned shoulder-width apart and arms relaxed at their sides. Safety was maintained via a harness attached to an overhead rail, which prevented falls without providing postural support. Participants were instructed to maintain balance without stepping if possible; however, no temporal cues regarding the perturbation onset were provided. Only a single trial was administered per participant to preserve the unpredictability of the perturbation timing, as previous lean-and-release studies have demonstrated that repeated exposure can lead to anticipatory adjustments [22]. The release was manually triggered by the examiner from a concealed location, and participants were not informed of the timing of the perturbation (Fig 2).

**Fig 2:**
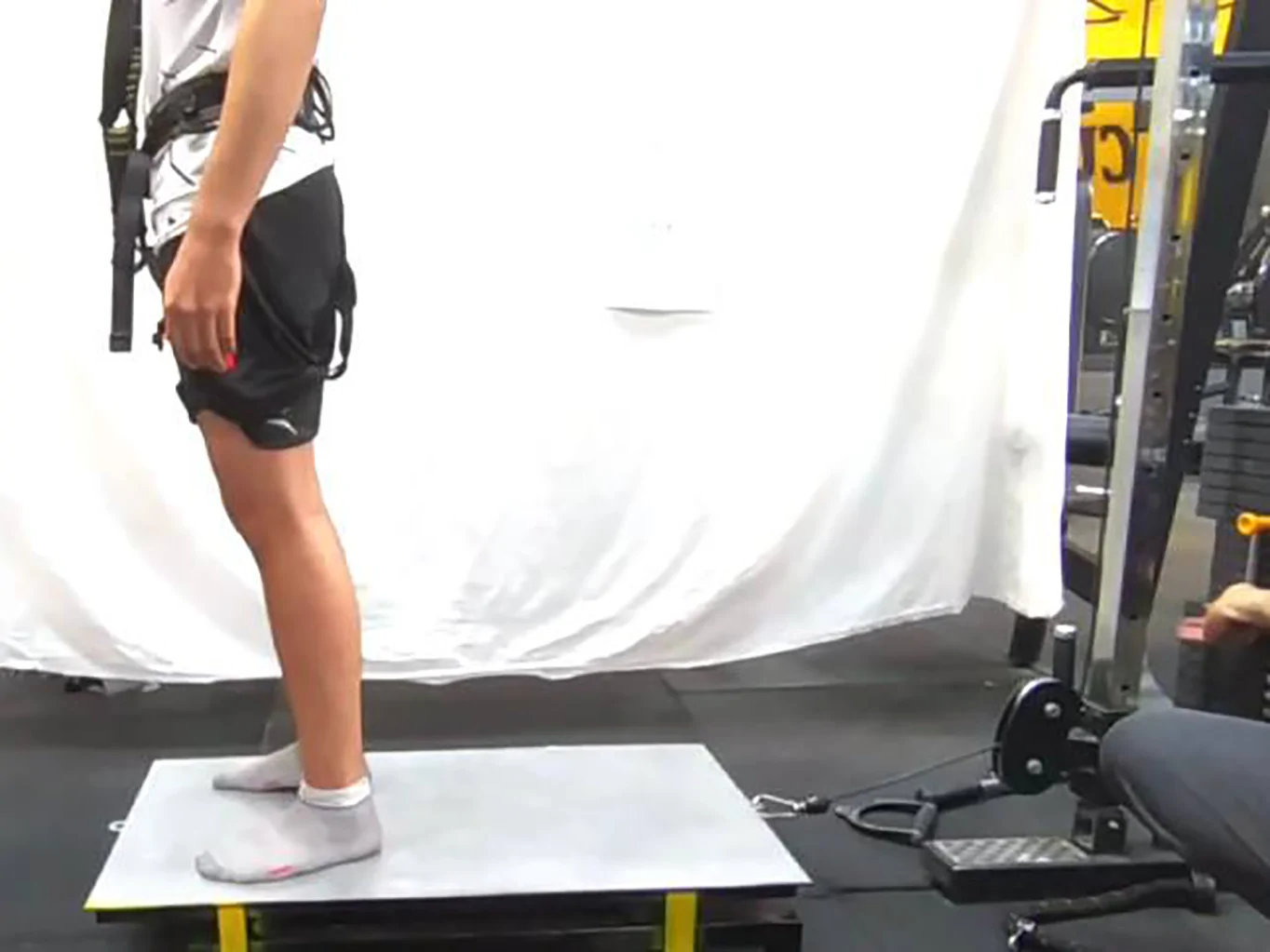
Participant positioning during the anterior perturbation assessment on the movable platform.

All experimental trials were captured in the sagittal view at a frame rate of 60 frames per second using a digital video camera positioned 3–4 meters lateral to the platform. The camera’s optical axis was meticulously aligned with the participant’s mid-sagittal body line to facilitate clear visualization of movements at the ankle, knee, hip, and trunk throughout each perturbation trial. The recorded videos were subsequently analyzed frame-by-frame using motion analysis software. Two independent raters, blinded to the participants’ DF-ROM status, independently reviewed each recording to identify the initial postural recovery response immediately following perturbation onset and classified the corresponding balance strategy based on observable kinematic patterns.

Postural responses were categorized according to established definitions of balance recovery strategies reported in the literature [3–5]. An ankle strategy was defined as a recovery response predominantly achieved through ankle plantarflexion or dorsiflexion, during which the body behaved largely as a rigid inverted pendulum with minimal observable movement at the hip or trunk. A hip strategy was characterized by recovery primarily driven by hip flexion or extension accompanied by substantial trunk motion, indicating greater proximal contribution relative to ankle torque. A stepping strategy was defined as any response involving elevation of one foot from the platform to execute a compensatory step, either forward or backward, thereby modifying the base of support relative to the center of mass. Classification was based on the first observable recovery movement following perturbation onset, with each trial assigned to a single strategy category. No predefined quantitative kinematic thresholds were applied; instead, classification was based on qualitative visual assessment of movement patterns according to established biomechanical definitions reported in the literature. Three trials exhibited combined ankle–hip characteristics and, according to the predefined classification protocol, were conservatively classified as hip strategy to maintain consistency with conventional postural control classifications. Any disagreements between raters were resolved through consensus discussions to ensure final agreement.

### Statistical analysis

Descriptive statistics were employed to characterize the study participants and to depict the distribution of balance strategies across different categories of ankle dorsiflexion. Categorical variables were presented as frequencies and percentages. The relationship between ankle dorsiflexion range of motion (classified as normal versus restricted) and the selection of balance strategies (ankle, hip, or stepping) in response to anterior perturbation was assessed using Pearson’s chi-square test of independence. Given the limited sample size and the occurrence of low expected cell frequencies, the assumptions underlying the chi-square test were not fully met; consequently, Fisher’s exact test was also conducted to obtain an exact measure of statistical significance. Additionally, likelihood ratio and linear-by-linear association tests were performed to complement the primary analysis. Statistical significance was predetermined at a threshold of p < 0.05. All analyses were carried out using the Statistical Package for the Social Sciences (SPSS, version 18.0; IBM Corp., Armonk, NY).

## Results

Table 1 presents the distribution of balance strategies across ankle dorsiflexion (DF) categories. Before consensus resolution, the two independent raters demonstrated substantial inter-rater agreement in the classification of postural recovery strategies (Cohen’s κ = 0.76). Trials demonstrating combined ankle–hip responses were classified as hip strategy in accordance with established postural control conventions. Following independent classification, any disagreements were resolved through consensus discussion, and the final consensus classifications were used for statistical analysis.

**Table 1:** Association between ankle dorsiflexion status and postural control strategy.

Variables		Postural control Strategies, n (%)				*p* value	
		Ankle	Hip	Stepping	Total (n)	Pearson Chi-square	Fisher’s Exact Test
Group	Normal Dorsiflexion ROM	2 (18.2%)	2 (18.2%)	7 (63.6%)	11	0.662	0.835
	Restricted Dorsiflexion ROM	3 (33.3%)	2 (22.2%)	4 (44.4%)	9		

Values are presented as number (percentage). Trials demonstrating combined ankle and hip responses were classified as hip strategy. Due to low expected cell counts, Fisher’s exact test was used to confirm the chi-square results.

In the normal DF group (n = 11), 2 participants (18.2%) adopted an ankle strategy, 2 (18.2%) a hip strategy, and 7 (63.6%) a stepping strategy. In the restricted DF group (n = 9), 3 participants (33.3%) used an ankle strategy, 2 (22.2%) a hip strategy, and 4 (44.4%) a stepping strategy. Trials demonstrating combined ankle–hip responses were classified as hip strategy in accordance with established postural control conventions. A Pearson chi-square test of independence (2 × 3 contingency table) revealed no statistically significant association between ankle dorsiflexion category (normal vs. restricted) and balance strategy selection, χ^2^ (2) = 0.826, p = 0.662. Because a high proportion of cells exhibited low expected frequencies (5 of 6 cells; 83.3% < 5), the assumptions of the chi-square test were violated. Therefore, an exact test was additionally performed, which likewise found no statistically significant association between dorsiflexion status and balance strategy selection (Fisher’s exact test, two-sided p = 0.835). Likelihood ratio and linear-by-linear association tests yielded consistent non-significant results. These findings indicate that no statistical evidence of an association between ankle dorsiflexion status and balance strategy selection was observed in this preliminary sample. However, given the small sample size and the low expected cell frequencies, the analysis had limited ability to detect small-to-moderate associations. Therefore, the non-significant findings should not be interpreted as evidence that no meaningful association exists.

## Discussion

The present investigation explored the relationship between ankle DF-ROM and reactive postural control mechanisms in response to anterior perturbations among healthy, physically active male adults. The findings revealed no significant association between ankle DF-ROM and the selection of postural recovery strategies following sudden anterior perturbations. Contrary to the initial hypothesis, both groups predominantly employed stepping strategies, accounting for 45%–64% of trials, with no observed increase in the utilization of hip strategies within the restricted dorsiflexion group. Notably, a marginally greater proportion of the normal dorsiflexion group adopted stepping strategies. These null findings contrast with prior research involving special populations, where mobility restrictions are frequently associated with shifts in postural strategies [14]. For instance, Kalantariyan et al. demonstrated that individuals with chronic ankle instability—a condition often characterized by dorsiflexion deficits—exhibited a greater reliance on hip movements and diminished ankle involvement during balance recovery [14]. Similarly, Basnett and colleagues identified a correlation between reduced DF-ROM and impaired anterior reach performance, indicative of compromised dynamic balance in athletes [15]. Furthermore, Hernández-Guillén et al. reported that ankle DF-ROM was the most robust predictor of dynamic balance among older adults [15]. Collectively, these studies imply that limited dorsiflexion may adversely affect “fixed-support” balance strategies.

Why did our cohort of healthy young adults fail to exhibit a comparable pattern? Several explanations may account for this observation. First, although the subgroup with restricted ROM in our study demonstrated an average ankle DF-ROM of approximately 16°, which is reduced relative to controls, this value is not markedly low. The perturbation applied may have imposed substantial postural demands that favored stepping responses in most instances, thereby potentially obscuring any subtle influence of DF-ROM. However, because the biomechanical characteristics of the perturbation were not directly quantified, this interpretation should be considered speculative. Indeed, the majority of trials across both groups culminated in a stepping response. One possible explanation is that the perturbation demands may have favored stepping responses over isolated ankle- or hip-dominant adjustments [3,22]. However, because platform displacement, velocity, acceleration, and applied pulling force were not directly measured, the biomechanical magnitude and loading characteristics of the perturbation cannot be established. Because perturbation magnitude was neither directly quantified nor experimentally manipulated in the present study, this interpretation should be considered speculative and warrants confirmation in future studies using instrumented and graded perturbation intensities. Consequently, the predominance of stepping responses may have reduced the sensitivity of the experimental paradigm to detect more subtle effects of ankle dorsiflexion ROM on fixed-support recovery strategies. When perturbation demands are sufficiently substantial to require rapid enlargement of the base of support, stepping may become the predominant recovery strategy and potentially mask differences attributable to ankle mobility alone. In the present study, however, whether the perturbation demands reached such a level cannot be determined because the relevant perturbation kinematics and applied force were not directly quantified. Therefore, the present findings should be interpreted within the context of the perturbation protocol and its associated task demands, recognizing that the biomechanical magnitude of the perturbation was not directly quantified.

Second, young adults characteristically execute a single, rapid recovery step following substantial perturbations, whereas older adults tend to employ multiple, smaller steps [23,24]. For example, Luchies et al. reported that young subjects predominantly took one recovery step under high perturbation conditions, while older subjects often required multiple steps [23]. Similarly, McIlroy and Maki observed that elderly individuals were twice as likely as younger counterparts to take additional steps to regain stability [24]. In our sample of physically fit individuals aged 18–25 years, stepping responses were immediate and generally limited to a single recovery step, which may have overshadowed any potential effects of ankle mobility on kinematic patterns due to the predominance of this recovery strategy. Third, it is plausible that our measurement of DF-ROM, assessed via a closed-chain lunge test with the knee flexed, did not fully encapsulate the functional demands of ankle mobility during perturbation recovery. Prior research suggests that even minimal ankle motion availability (on the order of 10–20°) suffices to engage an ankle strategy in highly trained individuals [3,4]. Once this threshold is surpassed, additional increases in ankle mobility may not confer further enhancement to an already adequate ankle strategy. Finally, the single-trial design of our study precluded the influence of practice or adaptation effects. All responses can be considered essentially naïve, thereby minimizing the impact of learning. However, some investigations into perturbation adaptation have demonstrated that repeated exposures can modify strategy selection [25]. We deliberately employed a single trial per participant to maintain unpredictability; consequently, our observations reflect only the immediate, initial reaction to perturbation. Therefore, the predominance of stepping responses observed in both groups should not be interpreted as evidence that ankle dorsiflexion has no influence on reactive balance control. Rather, it suggests that the demands imposed by the perturbation protocol may have reduced the sensitivity to detect more subtle effects of ankle mobility on strategy selection. However, because perturbation displacement, velocity, acceleration, and applied pulling force were not directly quantified, the extent to which the perturbation demands influenced strategy selection cannot be experimentally established from the present data.

Although no significant effect of ankle DF-ROM on strategy selection was observed in healthy young adults, this area warrants further investigation. Future research could benefit from exploring older or clinical populations, as well as systematically varying the magnitude of perturbations. Additionally, examining whether acute interventions, such as ankle stretching or mobilization, influence strategy utilization would provide valuable insights. Clinically, interventions aimed at increasing dorsiflexion—such as talocrural mobilizations—have been demonstrated to enhance dynamic balance in patients with ankle injuries [16,26], indicating a potential relationship between joint mobility and postural stability. Elucidating this relationship in healthy individuals may contribute to a better understanding of the role dorsiflexion plays in supporting reactive balance.

We recognize that the present study possesses certain limitations that warrant consideration. Firstly, as a pilot investigation, the sample size was relatively small, and the ankle DF-ROM was dichotomized for analytical purposes, potentially diminishing the sensitivity of the findings. The relatively small sample size also limits the statistical power of the study to detect associations of small or moderate magnitude. The a priori sample size calculation was based on a large effect size (Cohen’s *w* = 0.50); therefore, the present study may not have been sufficiently powered to detect smaller but potentially meaningful associations. Furthermore, we did not directly assess plantar flexor muscle strength or proprioceptive function, both of which may significantly impact the observed strategies. In addition, only a single perturbation trial was administered to each participant to preserve unpredictability and minimize anticipatory responses; however, this design increases the potential influence of random response variability and may not fully represent an individual’s typical reactive postural strategy, particularly in combination with the small sample size and qualitative strategy classification. Our classification approach was exclusively based on kinematic data; therefore, future research employing instrumented platforms or electromyography could provide a more nuanced characterization of subtle strategic components. In addition, the kinematic characteristics of the perturbation itself (e.g., platform acceleration, displacement, velocity, and applied force) were not directly instrumented, quantified, or formally validated. Although the mechanical configuration and loading protocol of the custom-built perturbation apparatus were standardized across all participants to promote consistent perturbation delivery, future studies should incorporate force sensors or motion capture systems to enable precise characterization, mechanical validation, and assessment of the reproducibility of perturbation intensity. Also, the perturbation paradigm utilized—a forward translation of a platform—may differ from alternative perturbation modalities, such as backward pushes or treadmill-induced perturbations, thereby limiting the generalizability of the results.

## Conclusion

In conclusion, ankle dorsiflexion ROM was not significantly associated with the reactive postural control strategy observed following the standardized anterior perturbation in this sample of healthy young adults. The predominance of stepping responses suggests that the perturbation imposed substantial postural demands; however, because displacement, velocity, acceleration, and applied force were not directly quantified, the specific biomechanical demands of the perturbation cannot be established. Therefore, the possibility that the perturbation demands reduced the sensitivity to detect an influence of ankle dorsiflexion ROM should be considered speculative rather than an experimentally demonstrated mechanism. These findings should be interpreted within the context of the present protocol and sample size and should not be taken as evidence that ankle dorsiflexion ROM has no influence on reactive postural control. Future studies should use instrumented, graded perturbations to determine whether the contribution of ankle dorsiflexion ROM varies with perturbation magnitude.
